# Influence of Habitat and Climate on the Spatial Distribution of Outbreaks of the *Hylesia metabus* Moth, Responsible for Lepidopterism, in Coastal French Guiana

**DOI:** 10.1007/s13744-026-01399-2

**Published:** 2026-06-18

**Authors:** Raphaël Fougeray, Isaline Orhon, Manon Denux, Romane Ibanez, Romane Leseur, Liliana Ballesteros-Mejia, Rodolphe Rougerie, Giacomo Sellan, Yi Moua, Mónica Arias, Melanie McClure

**Affiliations:** 1 Laboratoire Écologie, Évolution, Interactions des Systèmes Amazoniens (LEEISA), Université de Guyane, CNRS, IFREMER, Cayenne, France; 2 Direction de l’Expertise, DGD-REVE. Muséum National d’Histoire Naturelle, Paris, France; 3 CESAB, Centre de Synthèse et d’Analyse sur la Biodiversité, Montpellier, France; 4 Institut de Systématique, Évolution, Biodiversité (ISYEB), Muséum National d’Histoire Naturelle, CNRS, Sorbonne-Université, EPHE, Université des Antilles, Paris, France; 5 CIRAD, UMR EcoFoG (CNRS, AgroParisTech, INRAE, Université des Antilles, Université de Guyane), Campus Agronomique, Kourou, France; 6 UMR Espace-Dev, IRD, Univ Montpellier, Univ Guyane, Univ La Réunion, Univ Antilles, Univ Nouvelle-Calédonie, Montpellier, France; 7 UMR Espace-Dev, Université de Guyane, Cayenne, France; 8 UMR PHIM, CIRAD, INRAE, Institut Agro, IRD, Université Montpellier, Montpellier, France

**Keywords:** Ashen moth, Forest pest, Papillon cendre, Papillonite, Spatial heterogeneity, Species distribution model

## Abstract

Understanding the environmental context of insect outbreaks is crucial, particularly for pest species with significant impact on human health. The ashen moth *Hylesia metabus* is a generalist Lepidoptera, of which the females have urticating scales (*setae*) that cause severe dermatological reactions, and its outbreaks thus pose serious public health challenges along the coastal regions of Venezuela and French Guiana. Despite the species’ broad distribution throughout northern South America, outbreaks remain unpredictable and localized. Here, we explored factors that correlate with the spatial distribution of outbreaks by investigating 13 sites in French Guiana. We assessed forest structure, tree species composition, canopy cover, and avian predation rates in the field. Additionally, we performed species distribution modeling to explore the effect of climate. Outbreak-prone sites were associated with overall low tree densities, limited daily temperature variation, pronounced seasonal changes between the dry and rain seasons, and, surprisingly, higher avian predation rates which may be more indicative of favorable landscape structure. These conditions are more prevalent along the coast of French Guiana, contrasting sharply with the stable and diverse inland rainforest ecosystems where outbreaks are rarely reported. These findings highlight habitat features consistently associated with outbreaks and provide a first step toward identifying ecological conditions that may influence outbreak propensity and can inform future monitoring strategies under changing environmental conditions.

**Supplementary Information:**

The online version contains supplementary material available at 10.1007/s13744-026-01399-2.

## Introduction

A primary goal in insect ecology is to understand the mechanisms underlying spatial variability in the occurrence of insect outbreaks, particularly given their possible economic, ecological, and public health implications. Spatial heterogeneity is known to profoundly influence the dynamics, severity, and frequency of insect population fluctuations (Liebhold et al. 2004; Klemola et al. 2006), and this can be the result of abiotic factors, such as climate and habitat characteristics (Didham and Lawton 1999; Checa et al. 2014), and biotic factors, including host plant availability and predation pressure (Fortin and Mauffette 2001; Hughes et al. 2015). 

Understanding how these ecological factors shape the spatial occurrence of insect outbreaks is crucial, but predicting them in widely distributed generalist species remains difficult due to context-dependent responses to environmental variability and the limited availability of long-term monitoring data. But in the face of ongoing climate change, identifying the factors that determine why outbreaks occur only in certain regions or habitats becomes increasingly important. Indeed, warming climates are expected to alter the frequency, intensity, and spatial distribution of insect outbreaks, exacerbating their impacts on ecosystems and societies (Lehmann et al. 2020). A better understanding of the ecological mechanisms can therefore enable us to develop more effective management and mitigation strategies.

A notable example of a widely distributed, highly generalist insect pest is the ashen moth, *Hylesia metabus*, which occurs across diverse habitats within the northern Amazon basin, including both forested and coastal regions. Within this broad geographic range, non-cyclical and unpredictable outbreaks occur specifically in coastal areas of the Guiana Shield, particularly in French Guiana and Venezuela (Jourdain et al. 2012). Because *H. metabus* can cause severe cutaneous reactions, known as Lepidopterism (Luce et al. 2023), these outbreaks necessitate frequent safety measures including curfews and the use of large burners, a method developed to attract and destroy moths, and used in the townships of Sinnamary and Iracoubo (French Guiana) where outbreaks are especially problematic (CROPP 2015). Despite the health, social, and economic importance of these outbreaks, the ecology of *H. metabus* remains poorly known and has rarely been the subject of in-depth studies.

To understand why *H. metabus* outbreaks occur in some areas and not in others, and why some localities appear to be more prone to them, it is essential to identify the environmental factors that promote or constrain its population dynamics. In insects, abiotic factors, such as temperature, can directly affect larval growth and development, and influence geographic range and phenology (Kocsis and Hufnagel 2011; Checa et al. 2014). Structural landscape can also affect biotic interactions by determining community composition and spatial heterogeneity, which in turn can modulate the availability and quality of host plants (Fortin and Mauffette 2001) as well as predator community dynamics (Kareiva 1987). Together these factors could regulate *H. metabus* populations through density-dependent effects, ultimately shaping the spatial distribution and potential for outbreaks of this species (Dover and Settele 2008; Willems and Hill 2009).

This study examines the interplay between abiotic (climatic conditions and landscape structure) and biotic (host plant availability and avian predation rate) factors in shaping *H. metabus* distribution and outbreak intensity in French Guiana. We hypothesize that spatial variation of outbreaks is driven by differences in the habitat. Specifically, we test the relative influence of habitat composition (namely host plant availability, plant diversity, canopy cover, and avian predation rate), and climatic conditions (namely temperature and precipitation) on *H. metabus* distribution and abundance, using recent outbreak localities as a proxy.

## Materials and Methods

### Study Area

The study was carried out over 2 years (2020–2021) in French Guiana, an overseas department of France located on the northern coast of South America with a predominantly equatorial climate. Around 95% of French Guiana’s area is occupied by dense rainforest with very low human population density. The coast of French Guiana, where the majority of the population is found, is instead composed of a mosaic of savanna, mangrove, and coastal forest. It is on this coastal strip that moths of *H. metabus* can constitute a problem for public health. The most affected municipalities extend along the coastal road connecting Cayenne to Saint-Laurent-du-Maroni, via Kourou (CNEV 2011; Jourdain et al. 2012). Coastal populations of *H. metabus* appear to differ from the forest populations, with both periodic and non-cyclical outbreaks rather than constant population cycles, with some areas suffering from more frequent outbreaks of higher density (Vassal 1989; Jourdain et al. 2012; Ciminera 2017).

Although no standardized data currently exists on moth population densities or outbreak severity, we used a qualitative outbreak classification based on three types of indirect sources: (1) local and regional news reports between 2006 and 2021 that showed a higher frequency of reported events in a limited number of coastal municipalities, namely Sinnamary and Iracoubo, with several coastal and inland sites only sporadically affected (Table S1); (2) municipal actions to mitigate risks to public health (e.g., use of moth burners, implementation of curfews); and (3) structured and informal discussions with local residents. While these qualitative sources may be subject to biases, such as uneven media coverage, variation in public awareness, or differences in human population density, no significant correlation between human population size (Douriaud 2023) and outbreak propensity was found (Spearman’s *ρ* = −0.32, *p* = 0.29, *n* = 13). Importantly, outbreak propensity was defined exclusively from these indirect sources and independently of habitat type or vegetation structure.

**Fig. 1:**
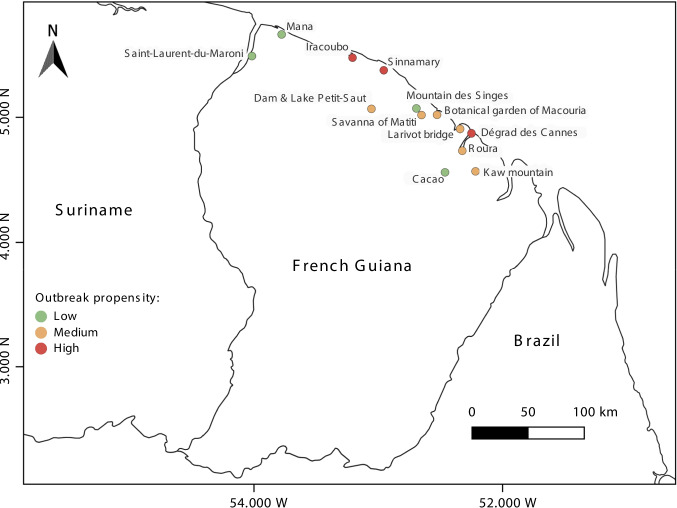
The spatial distribution of the 13 study sites; colors indicate categories of likelihood for outbreak of *Hylesia metabus* and negative human impact, based on news coverage, municipal records, and information from the public. Green indicates species presence but low outbreak reports number (*N* = 4: Saint-Laurent-du-Maroni, Mana, Mountain des Singes near Kourou, Cacao), yellow indicates a moderate number of outbreak reports, with occasional outbreaks of mild effects on the human population (*N* = 6: Dam & Lake Petit-Saut near Sinnamary, Savanna of Matiti near Macouria, Botanical garden of Macouria, Larivot bridge near Macouria, Roura, Kaw Mountain), and red indicates high and recurrent reports of severe outbreaks, including widespread Lepidopterism and need for costly municipal actions (*N *= 3: Iracoubo, Sinnamary, boat harbor of Dégrad des Cannes in Rémire-Montjoly)

For this study, 13 sites (Fig. 1) were selected in order to cover a gradient of perceived outbreaks, and categorized into three propensity classes: (1) presence of the species but low number of outbreak reports (*N* = 4 sites); (2) moderate number of reports, with occasional outbreaks of mild effects on the human population (*N* = 6 sites); (3) frequent reports of severe outbreaks, including widespread Lepidopterism and need for costly municipal actions (*N* = 3 sites).

### Habitat Composition

For each of the 13 sites, three line-intercept transects of 16 m were sampled. Line-intercept sampling is a type of transect whereby sampling quantifies the vegetation (i.e., in our case trees taller than 1 m) that intercepts a line segment (see Fig. S1 for a schematic overview of the method). Each tree was identified to the highest taxonomic level, either directly in the field, or by collecting voucher specimens for comparison with reference material in the herbarium collection of EcoFoG, in Kourou. The circumference of each tree was also measured at a height of 1.3 m from the ground, and tree height and the area of intercept were estimated. This method is widely used in forest inventories and has been shown to produce an accurate general overview of the habitat studied (Caratti 2006). Transect accuracy improves with increasing length of transects used, but 16-m transects have been found to be suitable in dense vegetation (Fraver et al. 2018). Here, the use of three transects of 16 m each, sufficiently spaced among them to avoid counting the same individual more than once and arranged randomly, allows to sample a total length of 48 m.

Taxonomic data of tree species found was used to calculate three diversity indices per transect: species richness, Shannon diversity, and Pielou’s evenness (using vegan R package; Oksanen et al. 2024; see ESM for details). Moreover, the basal area (Ge), a relatively easily measured surrogate of total biomass (McElhinny et al. 2005), was calculated using the circumference (C) of each tree with the formula $Ge=\frac{{C}^{2}}{4\pi }$. The total basal area for each transect was the sum of all the individual tree values, and the basal area specifically occupied by host plants within each transect was determined by summing the Ge values of all potential host plants (listed in Ciminera 2017; see also Table S2 which lists host plant species of *H. metabus*).

Total intercept length within a transect, and intercept length occupied by host plants, were the sum of intercept lengths of all trees and all host species intersecting with the transect, respectively (Fig. S1). In addition, canopy cover was measured by taking a photograph at each transect with a fish-eye lens (Samyang 7.5 mm lens/Olympus OM-D E-M5). Images were converted into binary color schemes (sky in white and vegetation in black) using ImageJ and the proportion of black pixels was calculated (see ESM for details). Finally, for each site, habitat type was determined based on a census and forest habitat cartography by the French National Forest Office (Guitet et al. 2015).

### Avian Predation Rate

To assess relative avian predation rates across habitats, artificial clay caterpillars (3 cm long and 0.5 cm in diameter) resembling common cryptic palatable caterpillars (see Fig. S2 for example) were made using light green plasticine (obtained by mixing modeling clay Van Aken yellow and plastilina Jovi green). This method was not intended to quantify predation on *H. metabus* larvae specifically, as these are chemically defended (Casafús et al. 2022) and specific predators are unknown, but rather to provide a standardized proxy of avian predation rate to compare between habitats.

Lure caterpillars were attached with a thin metal wire (diameter 0.5 mm) on branches 1.5–2 m from the ground and at least 2 m apart. One hundred artificial clay caterpillars were placed at each site, evenly distributed over each transect (33 or 34 caterpillars per transect), for an overall total of 1300 caterpillars. The caterpillars were left at each site for approximately 72 h. Predation marks on the malleable clay were categorized as either “avian” or “other/unknown” based on previous studies (see Low et al. 2014 and Fig. S3 for examples of predation marks) and only attacks by avian predators were used for further analyses. Although invertebrates and other vertebrates can be potential predators of caterpillars, it is difficult to distinguish real predation attacks from territorial aggression (for example by ants) and clay consumption. Artificial caterpillars not recovered were scored as missing and excluded from further analyses (*n* = 26).

As the data did not follow a normal distribution, a Kruskal-Wallis test was used to evaluate whether predation rates differed significantly among forest habitats using the rstatix R package (Kassambara 2020). Post-hoc pairwise comparisons were conducted using Dunn’s test (rstatix R package).

### Habitat Comparison

Given the categorical nature of the dependent variable (low, medium, and high outbreak propensity), a spatial generalized additive model (sGAM) with ordinal response structure was done using the mgcv R package. To avoid multicollinearity, the variance inflation factor (VIF) was calculated for all predictor variables, and those with a VIF greater than 5 were excluded from further analysis, as were those with sampling inconsistencies (see Table S3 for the full list of variables considered and those retained in the final models). The following factors were kept as explanatory variables: tree species richness (S), Pielou’s evenness index, predation rate, canopy cover (%), number of trees (as count data), amount of host plants (as count data), and total tree basal area (m^2^).

To account for potential spatial autocorrelation in the data, a bivariate smooth term on geographic coordinates was included using a Gaussian process spline with a Matérn covariance structure, which allows for flexible modeling of spatial dependence between observations as a function of geographic distance (Apparicio et al. 2025). Model selection was performed by fitting all possible additive combinations of predictor variables and comparing using the Akaike Information Criterion (AIC; Burnham and Anderson 2004). The best model (ΔAIC = 0) included predation rate, canopy cover, number of trees, and number of host plants as explanatory variables. Model diagnostics indicated a good fit with no violation of assumptions. Cross-validation revealed limited predictive performance, consistent with the small sample size and strong spatial structure of the dataset, indicating that the model is better suited for identifying ecological drivers than for predicting outbreak risk. Influence diagnostics identified a small number of observations with higher Cook’s distance, but sensitivity analyses confirmed that the main conclusions remained qualitatively unchanged (see Supplementary Material).

Finally, a principal component analysis (PCA - FactoMineR R Package; Lê et al. 2008) was performed to visually assess the distribution of sites based on the variables identified as significant in the sGAM. The PCA biplot, with outbreak categories shown in different colors, has 95% confidence ellipses to illustrate the clustering of sites.

### Species Distribution Model

To better understand how environmental conditions potentially influence the spatial occurrence of *H. metabus* in French Guiana, and to assess whether climatic suitability may help contextualize patterns of outbreak propensity, species distribution modeling (SDM) was used based on occurrence records and climate data collected between 1970 and 2000 (WorldClim 2 - Fick and Hijmans 2017). To compare the environmental niches occupied by *H. metabus* and its host plants, in order to assess the extent to which the moth occupies habitats that are ecologically analogous to those of multiple host species, models were also generated for three host plants in French Guiana (Table S2). Because *H. metabus* is a polyphagous species, all plant species reported as hosts in the literature were considered potential host plants (Table S2); the three species selected for SDM analyses therefore represent a non-exhaustive subset chosen based on frequent records and local relevance. Two mangrove species, *Avicennia germinans* (black mangrove) and *Laguncularia racemosa* (white mangrove), were included due to their reported association with *H. metabus* in mangrove ecosystems (Jourdain et al. 2012). Although *Rhizophora mangle* (red mangrove) is frequently reported as a primary host plant in Venezuela, in French Guiana, *H. metabus* seems to preferentially feed on *A. germinans* and *L. racemosa* (Jourdain et al. 2012). The third species, *Tapirira guianensis*, is a widespread tree found in both secondary and old-growth forests, selected because it was often reported as an important host by locals (*pers. obs.*). Furthermore, *T. guianensis* and *A. germinans* were commonly occurring host species in our transects (Table S2).

Occurrence data of *H. metabus* (*n* = 65) were obtained from multiple sources, including the Barcode of Life Data System (BOLD: www.boldsystems.org; Ratnasingham and Hebert 2007), the French National Museum of Natural History (MNHN - Paris, France), and field sampling data compiled from Ciminera (2017). Occurrence data for host plants (*A. germinans*: *n* = 63; *L. racemosa*: *n* = 41; *T. guianensis*: *n* = 63) were obtained from the Global Biodiversity Information Facility (GBIF). All spatial data were aligned using the WGS 84 (World Geodetic System 1984) - geographic coordinate system (EPSG:4326) at the 30 arc-seconds resolution (~1 km^2^) with the sf (Pebesma 2018) and raster (Hijmans 2023) R packages.

Climate variables were selected using a Pearson correlation matrix, with a threshold of 0.7 to exclude collinear variables, followed by a variance inflation factor (VIF) analysis with a cutoff value of < 10 (Guisan et al. 2017). The climatic variables selected were (1) Bio1: annual mean temperature; (2) Bio2: mean diurnal range; (3) Bio4: temperature seasonality, calculated as the standard deviation of monthly temperatures multiplied by 100, indicating the extent of temperature fluctuation across the year; (4) Bio18: precipitation during the warmest quarter; and (5) Bio19: precipitation during the coldest quarter.

To minimize modeling bias due to uneven sampling, we refined the selection of pseudo-absence points (i.e., background points in MaxEnt) using the target-group background method (Phillips et al. 2009; Barber et al. 2022). This method involves generating a background dataset that accurately reflects sampling effort by creating a density map derived from the recorded occurrence of groups collected using similar sampling methods within the same geographic region. Specifically, a two-dimensional Kernel density estimation was applied, following Barber et al. (2022), with occurrence data from Saturniidae, Sphingidae, and Noctuidae moth families in French Guiana (obtained from the MNHN and GBIF; *n* = 5821; see Fig. S4a for the resulting sampling-effort density surface). These families were chosen because their sampling methods—primarily light traps—match those used for collecting *H. metabus*. The resulting raster file, with the same spatial extent and grid resolution (30 arc-seconds) as the climatic variables, was rescaled to range from 1 to 20 following Elith et al. (2010) and assigned higher probabilities to background points drawn from areas with comparable sampling intensity to the *H. metabus* records. This approach effectively reduces the introduction of artifacts resulting from uneven survey efforts. The same approach was applied using Magnoliopsida occurrences (*n* = 75,262; Fig. S4b) to correct sampling bias in the host plant models. GBIF extractions (GBIF.org 2024) initially resulted in a total of 188,523 occurrences (moths and plants combined), before removing duplicates and excluding records with missing spatial coordinates or taxonomic identification using standard filtering procedures in R.

Initially, optimal modeling settings (feature classes and regularization multiplier) for each species were determined using the ENMeval R package (Muscarella et al. 2014), selecting the model configuration with the lowest corrected Akaike Information Criterion (AICc). To ensure methodological consistency across species, we retained the same model settings (features = Linear + Quadratic, regularization multiplier = 1) for all final models (see Table S4 for ENMeval model tuning). This configuration showed a ΔAICc < 2 for all species except *L. racemosa* (ΔAICc = 2.38), which was nevertheless considered acceptable given the high predictive performance of the model (AUC = 0.94). These settings were subsequently used to build final species distribution models using MaxEnt software (Phillips et al. 2006) with a tenfold cross-validation procedure. To assess the robustness of model predictions, we performed a sensitivity analysis for each species by comparing the final models to (i) models built using a uniform random background (i.e., without sampling bias correction) and (ii) models excluding the most influential environmental variable (see Supplementary Material). Finally, the predicted distribution of *H. metabus* was compared to those of its potential host plants through niche overlap analysis. Using the dismo R package (Hijmans et al. 2010), we calculated Schoener’s *D* and Hellinger-based *I* indices to quantify spatial similarity between species distribution models (Warren et al. 2008). Both indices range from 0 (no overlap) to 1 (complete overlap), but differ in their underlying assumptions and sensitivity. Schoener’s *D* measures absolute differences in predicted suitability across space, assuming that these values reflect relative habitat use. In contrast, the Hellinger-based *I* index treats model outputs strictly as probability distributions, without assuming a biological meaning, and is therefore more robust to extreme values and skewed predictions. By combining both metrics, we capture complementary aspects of niche similarity and minimize potential biases linked to any single interpretation of model outputs.

We further assessed niche divergence between *H. metabus* and each host plant by performing both equivalency and similarity tests using the ENMTools R package (Warren et al. 2021). Both tests use Schoener’s *D* and Hellinger-based *I* indices to quantify niche overlap, but differ in their null hypotheses. The niche equivalency test evaluates whether *H. metabus* and its host plants occupy ecologically equivalent niches, by comparing the observed overlap to that expected under random allocation of occurrences. The niche similarity test assesses whether *H. metabus*’ niche is better predicted by the environmental distribution of its host plants than expected by chance, thus testing whether they share similar environments beyond spatial proximity. Together, these tests provide a statistical framework to evaluate whether niche similarity reflects true ecological overlap or arises from environmental availability alone. All models used in these tests were generated with MaxEnt (feature class = LQ, regularization multiplier = 1). Due to package limitations, cross-validation was not applied; instead, each test was based on 99 permutations per species.

## Results

### Habitat Composition

The 13 sites sampled differed in forest structure and composition (see Table S5 for mean values of all variables for each site), but could be separated into four different categories (see Table 1 and Table S6 for mean values per forest habitat) based on the forest habitat cartography of the French National Forest Office (ONF; Guitet et al. 2015).

**Table 1:** Mean values (for the three transects) per site for selected ecological variables and diversity indices measured at 13 sites in French Guiana: values in bold indicate values greater than or equal to the median. The category outbreak propensity for each site is indicated in parentheses (low (1), medium (2), and high (3) outbreak propensity)

ID	Site	Number of trees	Number of host plants	Proportion of host plants	Canopy cover (%)	Tree species richness (S)	Forest habitat as per Guitet et al. 2015
1	Cacao (1)	**11.33**	2	0.16	**95**	**9.33**	Mid-altitude mountain forest
2	Mountain des singes (1)	**12**	0.33	0.05	89	**8.33**	Hill and valley forest
3	Mana (1)	6	**2.67**	**0.48**	**94**	**4.67**	Coastal forest (white sand forest)
4	Saint-Laurent-du-Maroni (1)	**8.67**	**3**	**0.35**	**96**	**5.33**	Coastal forest
5	Lake Petit-Saut (2)	**13**	0.33	0.04	**95**	**5.33**	Hill and valley forest
6	Kaw mountain (2)	**8.67**	**3**	**0.33**	**94**	**6**	Mid-altitude mountain forest
7	Roura (2)	**7**	**4.67**	**0.61**	79	3.67	Coastal forest
8	Bridge Larivot, Macouria (2)	5	**5**	**1**	88	2	Coastal forest (Mangroves)
9	Botanical garden, Macouria (2)	5.33	**3.33**	**0.56**	68	2.67	Savanna and coastal forest
10	Savanna of Matiti (2)	6	**2.33**	**0.39**	61	**4.67**	Savanna and coastal forest
11	Dégrad des Cannes (boat harbor, Rémire-Montjoly) (3)	**8**	**2.33**	0.31	**97**	4.33	Coastal forest
12	Iracoubo (3)	5.67	**2.33**	**0.38**	80	3	Savanna and coastal forest
13	Sinnamary (3)	6.33	1	0.15	**95**	**5.33**	Savanna and coastal forest
	Median	7	2.33	0.35	94	4.67	

The first category is coastal forests, which include five of our sites, spanning all three risk levels of outbreak. Although consisting of many different forest habitats, all are strips of lowland forest (> 20 m in altitude) that extend inland > 40 km along the coastline. This habitat category is characterized by low species diversity, high density of small stems, and low forest cover (Guitet et al. 2015). Notably, among the tree species encountered, about half (49%) were potential host plants to *H. metabus*. Within the coastal forest, the Mana and Larivot bridge sites were especially distinct. Mana is characterized by a white sand forest, with many endemic and rare species, generally uncommon elsewhere (Guitet et al. 2015). The Larivot bridge site consists of mangroves, which are periodically submerged by salty or brackish water, and have especially low diversity (it was the site with the lowest species richness) and tree species adapted to this unusual environment. Only two plant species were found in our Larivot transects, *A. germinans* and *L. racemosa*, and both are host plants for *H. metabus*.

The second category is a mix of savanna and coastal forest. This included four of our sites and they had a moderate to severe risk for *H. metabus* outbreaks. These were a mix of stands of coastal forests and grassy woodland characterized by trees sufficiently widely spaced so that the canopy did not close. Canopy cover of our transects was in fact the lowest for this habitat (76% cover vs 87% on average for all sites) and the transects had the least number of trees counted (5.83 per transect vs the 7.92 overall mean), a large percentage (37%) of which were potential host plants.

The third habitat category was the hill and valley forest, and it included two of our sites. These sites had fairly high tree density, and relatively abundant small stems. These forests are described as having dense undergrowth and mid to high canopy (30–35 m; Guitet et al. 2015). We found these sites to have a large number of trees (12.50 per transect vs the 7.92 overall mean) and a high species richness, but a very low percentage of these were potential hosts (4%). The fourth habitat category was the mid-altitudinal mountain forests and included two of our sites. These forests are described as having a high canopy (37 m) of irregular appearance, with high biomass, and where large trees are common and the undergrowth is very diverse (Guitet et al. 2015). Similar to forests of hill and valleys, the mid-altitudinal forest had a great stem density from different species, but a large percentage of them (23%) were found to be potential host plants, although they were classified as of similarly low to moderate risk for outbreak.

When comparing the different classes for outbreak propensity (Table 2), the sites most likely to suffer from frequent outbreaks (group 3, *n* = 3) were characterized by low tree count, whereas groups least likely to have outbreaks (group 1, *n* = 4) were characterized by high tree count and high diversity. Surprisingly, the highest proportion of host plants (43%) was found at sites of moderate risk (group 2, *n* = 6), although this difference appears to be solely due to the mangrove site where only two species, both potential hosts, were found.

**Table 2:** Mean values for some of the ecological variables and diversity indices measured at 13 sites in French Guiana per category of outbreak propensity (low (1), medium (2), and high (3) outbreak propensity)

Outbreak groups	Number of sites	Number of trees	Number of host plants	Proportion of host plants	Canopy cover (%)	Tree species richness (S)	Proportion of savannas and coastal forests
1	4	9.50	2	0.26	93.44	6.92	0.5
2	6	7.50	3.11	0.49	80.92	4.06	0.67
3	3	6.67	1.89	0.28	90.76	4.22	1

### Avian Predation Rate

Of the 1284 artificial caterpillars retrieved, 291 showed signs of predation, of which only 85 could be attributed to avian attacks, resulting in an average avian predation rate of 0.07. Although absolute predation rates were low overall, relative differences between sites were nevertheless observed, with values ranging from 0 to 0.17 (see Table S5 for means per locality). However, the Kruskal-Wallis test did not detect a significant overall effect of habitat type on predation rates (Statistic = 6.25, *p* = 0.1; but see Supplementary Material and Fig. S5 which shows differences between habitat types).

### Habitat Comparison

Spatial generalized additive model (sGAM) was fitted to account for the ordinal nature of the site categories for outbreak propensity, while controlling for spatial autocorrelation. The analysis revealed that only the predation rate, the canopy cover (%), and the number of trees were significantly associated with the potential for outbreak. Outbreak propensity increased with a higher predation rate (estimate = 177 ± 52, *z* = 3.38, *p* < 0.01), whereas it decreased with greater canopy cover (estimate = −0.30** ± **0.14, *z* = −2.17, *p* = 0.03) and with the number of trees (estimate = −3.94** ± **1.35, *z* = −2.91, *p* < 0.01). The number of host plants did not have a significant effect (*p* = 0.63). The spatial smoothing term was highly significant (*edf* = 5.77, *χ*^*2*^ = 331.4, *p* < 0.001), suggesting a strong spatial structure in outbreak patterns.

**Fig. 2:**
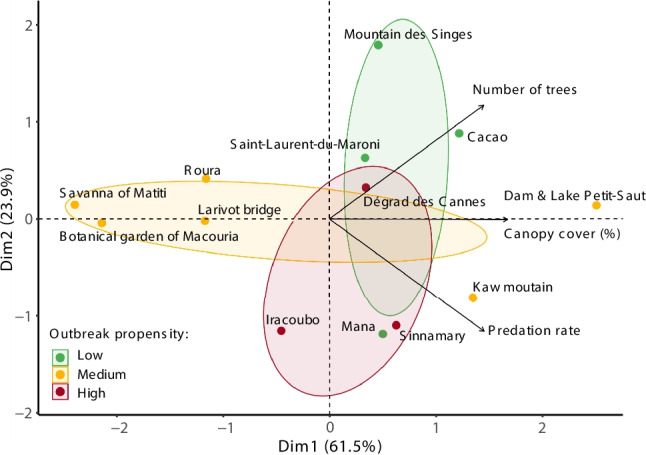
Principal component analysis (PCA) grouping sites as a function of outbreak propensity, and showing the effect of predation rate, number of trees, and canopy cover

The final model explained 99.4% of the deviance, a high value that is likely the result of the small number of sites used, an ordinal response variable, and a strong spatial structuring of outbreak propensity captured by the spatial smoothing term. That said, the principal component analysis (PCA) revealed clear differences among sites in relation to outbreak propensity (Fig. 2). Sites with low outbreak propensity were characterized by structurally complex habitats, with higher tree density and canopy cover, and generally low avian predation rates. Sites with intermediate outbreak propensity encompassed a broader range of habitat conditions, showing variable forest structure and predation rates. In contrast, sites associated with frequent outbreaks are characterized by habitats with low tree density (i.e., both reduced tree count and reduced canopy cover) and high predation rate (Fig. 2, see Supplementary Material for details).

### Species Distribution Model

**Fig. 3:**
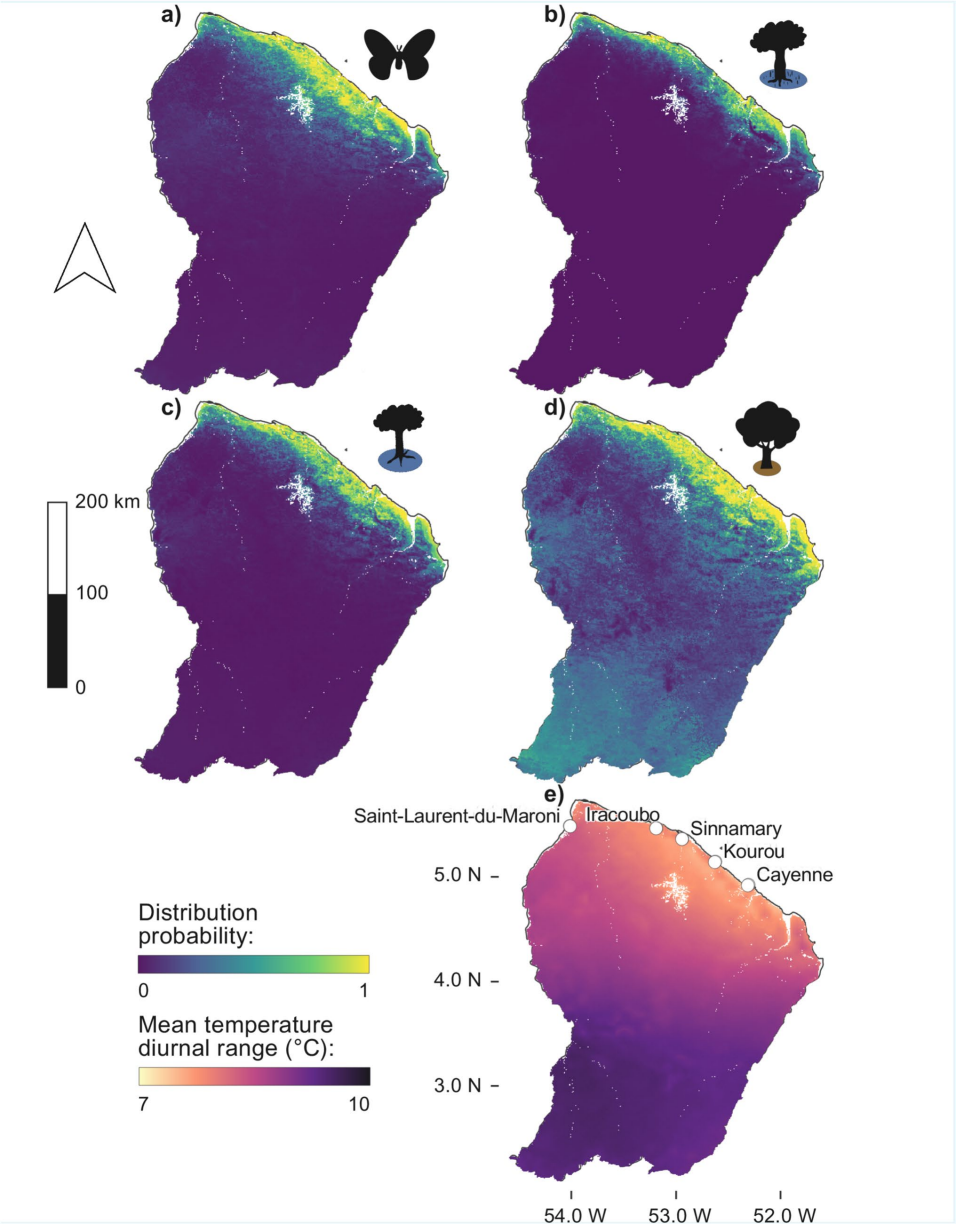
Species distribution model for **a** *Hylesia metabus*, **b** *Avicennia germinans*, **c** *Laguncularia racemosa*, and **d** *Tapirira guianensis,* with likelihood ranging from 0 (blue) to 1 (yellow). The mean diurnal temperature range from WorldClim (Fick and Hijmans 2017) is shown in **e**, ranging from 7 (orange) to 10 (purple)

Species distribution models (SDMs) for *H. metabus* and its host plants show reasonable or high performance with AUC values greater than 0.7 (*T. guianensis*) or 0.9 (*H. metabus*, *A. germinans*, *L. racemosa*) as shown in Table 3 (Swets 1988; Peterson et al. 2011). The models showed a higher probability for the presence of the moth *H. metabus* along the coastline (Fig. 3a and Fig. S6 that focuses on the coast). Although *H. metabus* has been found to occur inland, these habitats appear less suitable for the species based on both collecting data and the distribution model.

**Table 3:** Results of SDMs with permutation importance (PI) of each variable, for the moth *Hylesia metabus* and three of its common host plants; *Avicennia germinans*, *Laguncularia racemosa*, and* Tapirira guianensis*

	*H. metabus*	*A. germinans*	*L. racemosa*	*T. guianensis*
AUC	0.92	0.97	0.93	0.76
Bio1—annual mean temperature	0.5	11.7	3.3	1
Bio2—mean diurnal range	88.9	79.3	69.8	64.8
Bio4—temperature seasonality	0	1.2	0.5	0.3
Bio18—precipitation of warmest quarter	10	4.9	26	31.3
Bio19—precipitation of coldest quarter	0.5	3	0.5	2.7
Spatial overlap with the *H. metabus* model				
Hellinger’s *I*	1	0.87	0.98	0.89
Schoener’s *D*	1	0.61	0.84	0.60

The areas with the highest probability of occurrence are situated along the coast between Iracoubo and a little past Cayenne and Rémire-Montjoly. These areas were also classified as being at moderate to high risk of outbreaks based on our classification. Although some occurrence is predicted on the eastern coast, around Saint-Laurent-du-Maroni and Mana, we have found this region to actually be at low outbreak risk. The climatic variable that contributed the most to the predicted distribution of *H. metabus* was the mean diurnal temperature range (PI = 88.9). The host plants *A. germinans* and *L. racemosa* were also predicted to occur along the coast (Fig. 3b, c), consistent with their ecological restriction to mangrove habitats. Their distributions were also primarily influenced by the mean diurnal temperature range (PI = 79.3 and 69.8, respectively), with additional contributions from the annual mean temperature for *A. germinans* (PI = 11.7) and the precipitation of the warmest quarter for *L. racemosa* (PI = 26). The host plant *T. guianensis* had a broader predicted range (Fig. 3d), with its distribution primarily associated with the mean diurnal temperature range (PI = 64.8) and precipitation of the warmest quarter (PI = 31.3).

The highest similarity for spatial overlap was found with *L. racemosa* (*D* = 0.84; *I* = 0.98), followed by *T. guianensis* (*D* = 0.60; *I* = 0.89) and *A. germinans* (*D* = 0.61; *I* = 0.87). These values indicate moderate to high spatial overlap between the moth’s predicted distribution and those of its potential host plants, especially *L. racemosa*, which shows near-complete similarity based on the *I* index (Table 3).

**Fig. 4:**
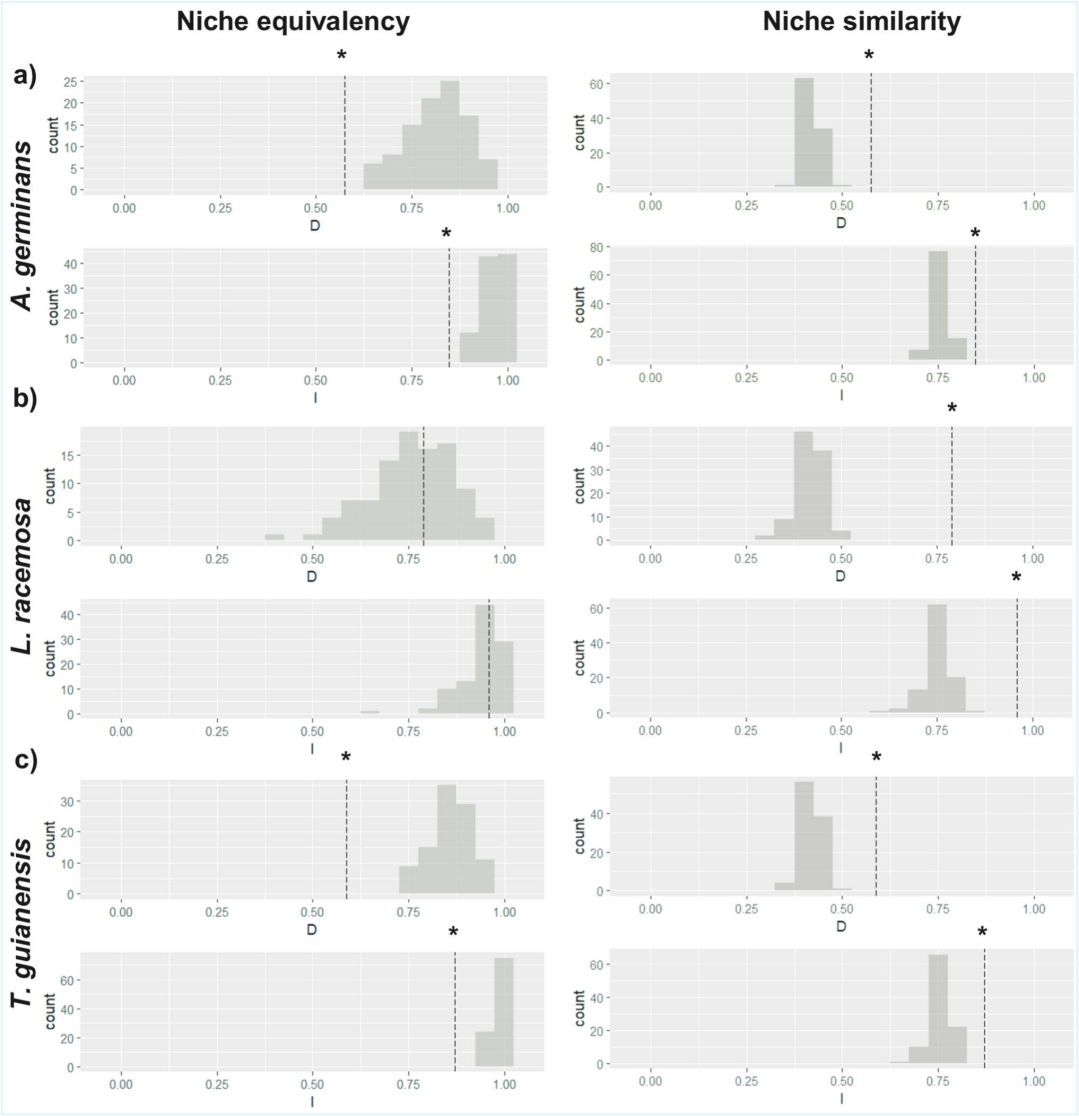
Niche equivalency and similarity tests between *Hylesia metabus* and its host plants. Each panel shows the observed niche overlap between *Hylesia metabus* and a host plant (dashed line) compared to the distribution of overlap values from 99 random permutations (pink histograms). Tests were conducted using Schoener’s *D* (upper plots per species, *D*) and Hellinger-based *I* indices (lower plots per species, I). Asterisks (*) indicate significant differences for the niche equivalency test and significantly higher similarity than expected under the null model for the niche similarity test (*p* < 0.05). **a** *Avicennia germinans*; **b** *Laguncularia racemosa*; **c** *Tapirira guianensis*

However, niche equivalency and niche similarity tests (Fig. 4) revealed important distinctions. The niche equivalency test did not detect significant differences between that of *H. metabus* and *L. racemosa* (*p*-values > 0.05), suggesting that their ecological niches may be equivalent. In contrast, those of *H. metabus* and *A. germinans*, as well as *T. guianensis*, were significantly different (*p* = 0.01 for both D and I), indicating that their niches are not statistically equivalent. Niche similarity tests indicated significant niche similarity between *H. metabus* and all three host plants (*p* = 0.01 for *D* and *I* in each case), suggesting that despite non-equivalent niches, the moth consistently occupies environments similar to those of its preferred hosts.

## Discussion

Habitat heterogeneity can affect where insect pest outbreaks occur (Liebhold et al. 2004; Klemola et al. 2006), and understanding how these ecological factors shape these occurrences is crucial for predicting outbreak-prone sites, lessening their impacts, and guiding future monitoring efforts. Here, we attempted to determine the biotic and abiotic factors that may explain the spatial differences in the non-cyclical outbreaks of *H. metabus* in French Guiana, a moth responsible for Lepidopterism. Despite the substantial public health impact of *H. metabus* outbreaks, their ecology remains poorly documented in French Guiana. As a result, descriptive and integrative approaches represent a necessary first step to characterize outbreak-associated environments and to identify hypotheses that can be tested through future targeted and experimental studies.

Because standardized long-term abundance data for *H. metabus* in French Guiana are not available, outbreak propensity was inferred from indirect sources such as media reports, municipal intervention, and public perception. While this approach may be influenced by reporting intensity and human population density, it provides a good approximation of outbreak impact, particularly in the context of public health. Importantly, this metric captures not only biological population increases but also their societal relevance. Nevertheless, future studies relying on long-term standardized monitoring tools (e.g., light traps) would be useful.

Here, we found that sites categorized as experiencing more frequent or severe outbreaks were found to be characterized by lower tree densities (i.e., reduced number of trees and overall canopy cover) and high avian predation rate. While the model showed limited predictive performance in cross-validation, likely due to the small sample size, it nevertheless provides valuable insight into the ecological factors associated with sites already identified as having different levels of outbreak propensity. These sites were more likely to occur in habitats consisting of coastal forests (including, but not limited to, mangroves) and savannas, which were generally correlated with low diversity and low overall tree count, of which a fairly high proportion were host plants. These habitats were also more likely to have pioneer plant species, many of which are potential hosts (e.g., *Cecropia obtusa*, *T. guianensis*), although host plant density was not correlated with outbreak propensity, suggesting that this may not be a limiting factor. This is also consistent with other studies that have found that polyphagous insects like *H. metabus* prefer and/or do better in fragmented habitats of low spatial heterogeneity and complexity (Tscharntke et al. 2002; Benedick et al. 2006).

Surprisingly, outbreak-prone sites were correlated with high avian predation rate, and may have correlated more with habitat types than outbreak risk; for example, outbreak-prone sites of coastal forests had relatively high predation rates, but others like the mangroves and savannas did not. This heterogeneity suggests that avian predation is not a robust predictor of outbreak propensity per se, but rather reflects broader differences among habitat types. It is worth noting that larvae of *Hylesia* possess stinging defenses and exhibit gregarious behavior which, despite being cryptic, may limit their vulnerability to avian predators (but see, e.g., Barbaro and Battisti 2011).

As such, the correlation between high avian predation rate and outbreak propensity may instead be the result of broader habitat characteristics, such as vegetation openness and edge density, which may covary with outbreak-prone environments (Kareiva 1987; Baggio et al. 2010). For instance, in the forest tent caterpillar *Malacosoma disstria*, the sensitivity of outbreak duration to changes in forest structure has been hypothesized to reflect reduced effectiveness of natural enemies such as parasitoids and pathogens in fragmented forests (Roland and Kauppp 1995; Roland and Taylor 1997). However, this remains to be tested in this system and although parasitoids and diseases of *H. metabus* were not investigated here, future work should compare mortality rate and causes between coastal and inland habitats, as these may also explain differences in their population dynamics.

As for climatic conditions, species distribution modeling (SDM) identified mean diurnal temperature range and precipitation during the warmest quarter as key predictors of *H. metabus* occurrence. In particular, the coast of French Guiana, characterized by more of a tropical monsoon climate with a distinct dry season and minimal diurnal temperature fluctuations, was predicted to be the most favorable. In contrast, the inland forests, where outbreaks have rarely been reported, are characterized by a tropical rainforest climate, with greater climatic stability and less pronounced seasonality (Beck et al. 2023). The strong influence of mean diurnal temperature range was further supported by sensitivity analyses, as excluding this variable substantially altered model predictions, highlighting its central role in shaping the predicted distribution of the species. Notably, this variable appears to follow a clear spatial gradient from the coast to inland areas, with lower diurnal temperature variation along the coastline and higher variability inland, mirroring the predicted suitability pattern. Importantly, this pattern is unlikely to be driven by spatial sampling bias, which could be expected to be higher along the more densely populated coastal areas. Models with and without bias correction showed minimal differences in both predictive performance and spatial predictions, suggesting that the observed coastal suitability does not result from uneven sampling effort.

Interestingly, SDM predictions also indicated high climatic suitability in some northwestern coastal areas (e.g., Mana and Saint-Laurent-du-Maroni) that were classified as low risk based on outbreak reports. This suggests that climatic suitability alone may be insufficient, and that additional factors such as local landscape configuration, biotic regulation, or differences in human habitat use may modulate local outbreak propensity. Importantly, this mismatch between predicted suitability and observed outbreak propensity is unlikely to be explained by sampling bias, as models with and without bias correction produced highly similar spatial predictions. Although the three host plants used for the distribution models are known to be common and frequently exploited by *H. metabus* (Jourdain et al. 2012), only the spatial distribution of *L. racemosa* closely matched that of the moth. Nevertheless, our results revealed significant ecological similarities between the niches occupied by *H. metabus* and these three host plants, suggesting that the moth exploits a broader ecological niche than would be inferred solely from spatial overlap with a single host plant species (*L. racemosa*). This indicates that *H. metabus* likely occupies habitats ecologically analogous to multiple host species, even when their geographic co-occurrence is limited, suggesting ecological flexibility in the species allowing it to efficiently use resources distributed heterogeneously in space.

Although for specialist insects, host plant availability can be an important driver of herbivore dynamics (Opedal et al. 2020), for the polyphagous *H. metabus*, overall abundance of host plants was not found to be correlated with outbreak-prone sites. This might be because host plant abundance alone provides an incomplete picture of resource limitation for a polyphagous herbivore. Factors such as host plant species composition, nutritional quality, secondary chemistry, and phenological synchrony with larval development may all influence habitat suitability and outbreak propensity, but were not explicitly addressed in this study. Likewise, the restriction of the SDM comparison to three representative host species was intended to capture broad ecological affinities rather than the full complexity of host use by *H. metabus*. Nevertheless, *H. metabus* appears to consistently occupy environments that are similar to some of its host plants, implying a shared preference for certain climatic conditions. While the availability and distribution of host plants likely play a key role in shaping the moth’s range, climatic variables appear to be even more influential in determining its overall distribution and outbreak propensity. As global warming and increased land-use change continue to modify habitats, the potential future expansion or contraction of *H. metabus*’ range warrants further investigation (Lehmann et al. 2020).

In conclusion, our results suggest that reported *H. metabus* outbreaks are preferentially associated with open, coastal habitats characterized by low tree density, limited diurnal temperature variation, and pronounced seasonality (i.e., marked dry and wet seasons). These characteristics are more likely to occur on the coast, and contrast markedly with the seasonally stable (i.e., minimal differences between seasons) and diverse inland rainforest ecosystems, where outbreaks have rarely been reported. Avian predation appears to be an unlikely mechanistic driver of outbreaks and may instead reflect broader landscape structure such as fragmentation and edge effect. Future studies should also investigate the potential effect of other natural enemies, such as parasitoids and diseases, which may be more negatively affected by habitat types and fragmentation, and which are more common along the coast, as well as host plant preferences and suitability. Targeted monitoring of both *H. metabus* populations and their natural enemies, combined with landscape management strategies that preserve forest complexity and reduce fragmentation, may help mitigate potential future risks. Long-term studies integrating ecological, climatic, and epidemiological data may be necessary to fully understand and anticipate the drivers of pest outbreak emergence in tropical systems.

## Supplementary Information

Below is the link to the electronic supplementary material.

ESM1 (DOCX 5.04 MB)

## Data Availability

Data are available from Zenodo 10.5281/zenodo.15414490 (Fougeray et al. 2026).
