# Supplementary material for: Influence of Habitat and Climate on the Spatial Distribution of Outbreaks of the Hylesia metabus Moth, Responsible for Lepidopterism, in Coastal French Guiana
Source: Neotrop Entomol. 2026 Jun 18;55(1):60. doi: 10.1007/s13744-026-01399-2 (PMC13279682; doi:10.1007/s13744-026-01399-2)
Supplement: Supplementary file 1 — (DOCX 5.04 MB) [file 13744_2026_1399_MOESM1_ESM.docx]

# **Electronic Supplementary information for**

**Influence of habitat and climate on the spatial distribution of outbreaks of the Hylesia metabus moth, responsible for Lepidopterism, in coastal French Guiana**

Raphaël Fougeray^1^, Isaline Orhon^1^, Manon Denux^1^, Romane Ibanez^1^, Romane Leseur^1^, Liliana Ballesteros-Mejia^2,3^, Rodolphe Rougerie^4^, Giacomo Sellan^5^, Yi Moua^6,7^, Mónica Arias ^8^† and Melanie McClure ^1^†*

^1^Laboratoire Écologie, Évolution, Interactions des Systèmes Amazoniens (LEEISA), Université de Guyane, CNRS, IFREMER, Cayenne, France

^2^Direction de l’Expertise, DGD-REVE. Muséum National d’Histoire Naturelle, Paris, France

^3^CESAB, Centre de Synthèse et d’Analyse sur la Biodiversité, Montpellier, France

^4^Institut de Systématique, Évolution, Biodiversité (ISYEB), Muséum National d’Histoire Naturelle, CNRS, , Sorbonne-Université, EPHE, Université des Antilles, Paris, France

^5^CIRAD, UMR EcoFoG (CNRS, AgroParisTech, INRAE, Université des Antilles, Université de Guyane), Campus Agronomique, Kourou, France

^6^UMR Espace-Dev, IRD, Univ Montpellier, Univ Guyane, Univ La Réunion, Univ Antilles, Univ Nouvelle-Calédonie, Montpellier, France

^7^UMR Espace-Dev, Univ Guyane, Cayenne, France

^8^UMR PHIM, CIRAD, INRAE, Institut Agro, IRD, Univ Montpellier, Montpellier, France

†Co-last authors: Mónica Arias & Melanie McClure

*Corresponding author E-mail: [melanie.mcclure@cnrs.fr](mailto:melanie.mcclure@cnrs.fr)

## **French abstract**

Comprendre comment les facteurs écologiques influencent l’occurrence spatiale des pullulations d’insectes est essentiel, en particulier pour les espèces nuisibles ayant un impact significatif sur la santé humaine. Le papillon cendre *Hylesia metabus* est un Lépidoptère généraliste, dont les femelles sont dotées d’écailles (*setae*) urticantes causant de sévères réactions dermatologiques, et les pullulations de ce papillon posent ainsi de sérieux problèmes de santé publique le long des côtes du Venezuela et de la Guyane française. Malgré sa large distribution dans le nord de l’Amérique du Sud, les pullulations restent imprévisibles et localisées. Dans cette étude, nous avons exploré les facteurs associés à la distribution spatiale des pullulations en examinant 13 sites en Guyane française. Nous avons évalué la structure forestière, la composition spécifique des arbres, la couverture forestière, ainsi que la pression de prédation aviaire. Nous avons également utilisé des modèles de distribution d’espèces afin d'examiner l’effet des conditions climatiques. Les sites plus à risques se caractérisent par une faible densité d’arbres, un taux de prédation élevée, des variations de température quotidienne minimales et des changements saisonniers marqués. Ces conditions sont plus fréquentes le long du littoral guyanais, contrairement aux écosystèmes forestiers intérieurs, plus stables et diversifiés, où les pullulations sont rares, voire absentes. Ces résultats mettent en évidence les caractéristiques des habitats associées aux pullulations, constituant une première étape vers la mise en place de stratégies de surveillance et de mitigations futures dans un contexte de changement environnemental.

**Spanish abstract**

Comprender el contexto ambiental de las pululaciones de insectos es crucial, especialemnte para aquellas especies plaga que tienen un impacto significativo en la salud humana. La palometa peluda Hylesia metabus es un lepidóptero generalista cuyas hembras adultas poseen pelos urticantes respoensables de reacciones dermatológicas graves. Las pululaciones de H. metabus imponen serios desafíos para la salud pública en las regiones costeras de Venezuela y la Guayana Francesa. A pesar de su amplia distribución en el norte de Sudamérica, las pululaciones siguen siendo impredecibles y localizadas. En este estudio, exploramos potenciales factores que se pueden correlacionar con la distribución espacial reciente de las pululaciones de H. metabus mediante la investigación de 13 sitios en la Guayana Francesa. Evaluamos la estructura del bosque, la composición de las especies arbóreas, la cobertura del dosel y las tasas de depredación aviar en el campo. Además, construimos modelos de distribución de especies para explorar el efecto del clima. Los sitios propensos a pululaciones se asociaron con bajas densidades de árboles en geenral, una variación térmica diaria limitada, cambios estacionales pronunciados entre la estación seca y la estación lluviosa y, sorprendentemente, mayores tasas de depredación aviar, lo que podría ser sobretodo un indicador de la estructura del paisaje. Estas condiciones son más frecuentes a lo largo de la costa de la Guayana Francesa, en marcado contraste con los ecosistemas de selva tropical del interior, estables y diversos, donde rara vez se reportan pululaciones. Estos hallazgos resaltan las características del hábitat asociadas consistentemente con las ubicaciones de pululaciones recientes, brindan un primer paso para identificar las condiciones ecológicas que pueden influir en la propensión a las pululaciones y pueden fundamentar futuras estrategias de monitoreo bajo condiciones ambientales cambiantes.

## **Materials and Methods**

### Risk of Hylesia metabus outbreaks

To assess the risk of *H. metabus* outbreaks along the coast, we categorized sites based on local outbreak records. This assessment was conducted by reviewing local news articles, identifying municipal needs related to the medical and social consequences of outbreaks, and through informal discussions with members of the general public in the affected areas. The review focused on articles from *France-Guyane*, *Guyane la 1ère*, and available reports from the *Cellule Régionale Permanente d’Observation et de Prévention de la Papillonite en Guyane* (CROPP; a regional service to help predict and prevent outbreaks). To avoid overestimating outbreak frequency by counting multiple mentions of the same event, only one outbreak report per locality was recorded when they occurred within a one-month interval. Once outbreak propensity of the different sites was determined based on these indirect sources, they were compared to the number of inhabitants to confirm that there was no perception bias, with areas of higher human density showing greater media activity irrespective of moth density. No such correlation was found. An inventory of the collected data and available documents is provided in Table S1. Additional details, including links to relevant press articles (in French), are available in the Zenodo archive <https://doi.org/10.5281/zenodo.15414490>.

| **Table S1** Number of *Hylesia metabus* outbreak reports in the local press and public information bulletins in French Guiana from 2006 to 2021. Reports occurring within a one-month interval in the same locality were considered as a single event to avoid duplication. | |
| --- | --- |
| **City** | **Number of times *H. metabus* were reported** |
| Sinnamary | 10 |
| Iracoubo | 9 |
| Rémire-Montjoly | 7 |
| Matoury | 5 |
| Roura | 5 |
| Cayenne | 4 |
| Kourou | 3 |
| Macouria | 2 |
| Cacao | 1 |
| Saint-Laurent-du-Maroni | 1 |
| Mana | 1 |

### Line intercept transect

At each study site, three 16-meter line-intercept transects were used to sample tree communities. This method quantifies vegetation by recording the length of plant material (trunks, branches, leaves) intersecting the transect line (Fig. S1). For each tree intersecting the line, species identity, circumference at 1.3 m from the ground, and intercept length were recorded. This technique, commonly used in forest inventories, provides reliable estimates of vegetation structure, especially in dense environments (Caratti 2006; Fraver et al. 2018).

| 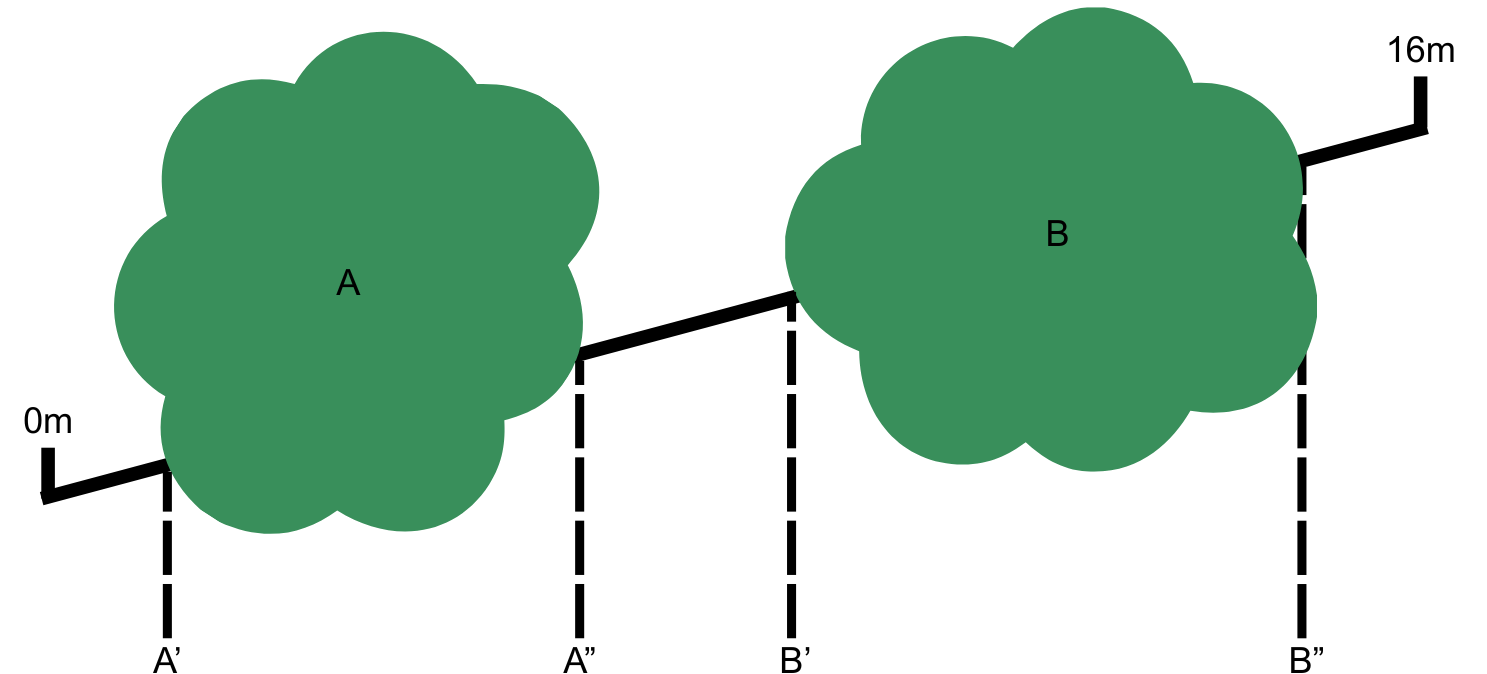 |
| --- |
| **Fig. S1** Schematic representation of a line-intercept transect: A’-A” and B’-B” show the measures for the intercept lengths of tree A and tree B (in green) intersecting the transect |

###

### Biodiversity indices

For each line-intercept transect, three diversity indices were calculated: species richness, Shannon and Piélou. Species richness ($S$) was calculated as the total number of species observed within each sampling unit. This metric provides a simple count of the unique taxa present, without accounting for their relative abundances. Shannon diversity index ($H'$) was calculated using the formula:

$$H' = -\sum_{i = 1}^{S} p_{i}ln(p_{i})$$

where $S$ is the total number of species, and $p_{i}$ is the proportional abundance of the $i$-th species in the community (i.e., the number of individuals of species $i$ divided by the total number of individuals across all species). The Shannon index accounts for both species richness and the evenness of species abundances, assigning higher values to communities with a more equitable distribution of individuals among species. Pielou’s evenness index ($J'$) was calculated as:

$$J' = \frac{H'}{ln(S)}$$

where $H'$ is the Shannon diversity index and $S$ is the species richness. Pielou's index provides a standardized measure of evenness, ranging from 0 (high dominance by a few species) to 1 (perfectly even distribution of individuals among species). All calculations were performed in R v.4.4.0. using the vegan package (Oksanen et al. 2024).

### List of host plants

*Hylesia metabus* is a polyphagous moth whose larvae feed on a wide variety of plant species (Table S2). Approximately 32 host plants have been documented (see Ciminera 2017 and references therein), spanning 20 different families, which reflects the ecological flexibility of this species. The list includes both native and introduced plants, ranging from common tropical trees such as *Cecropia obtusa* (Urticaceae) and *Virola sebifera* (Myristicaceae) to cultivated or economically important species like *Theobroma cacao* (Malvaceae), *Mangifera indica* (Anacardiaceae), and *Psidium guajava* (Myrtaceae).

### Measure of canopy cover using ImageJ

The forest cover at each transect was assessed using photographs of the canopy taken with a fisheye lens (Samyang 7.5mm lens / Olympus OM-D E-M5). These photographs were imported into the software ImageJ, where a mask was created using the color threshold tool by reducing the maximum brightness value, such that the forest appeared darker than the sky. The images were then converted into binary format using the "make binary" tool, with the masked areas (forest) represented as white pixels and the remaining areas (sky) as black pixels. The percentage of white pixels in the image was calculated using the "measurement" tool, providing the percentage of forest cover.

| **Table S2** Host plants of *Hylesia metabus*, as listed in Ciminera 2017, with the number of individuals found in the transects (identified to the species when possible, but also included when identified to the genus; empty cells correspond to 0). | | |
| --- | --- | --- |
| **Family** | **Species** | **Number of individuals in the transects** |
| Acanthaceae | *Avicennia germinans* | 8 |
| Anacardiaceae | *Anacardium occidentale* |  |
|  | *Mangifera indica* |  |
|  | *Tapirira guianensis* | 10 |
| Annonaceae | *Annona squamosa* | 34 |
|  | *Guatteria sp.* |  |
| Bignoniaceae | *Jacaranda copaia* |  |
| Bixaceae | *Bixa orellana* |  |
| Combretaceae | *Laguncularia racemosa* |  |
|  | *Terminalia catappa* |  |
| Euphorbiaceae | *Croton matourensis* |  |
|  | *Hura crepitans* |  |
|  | *Maprounea guianensis* | 3 |
|  | *Sapium glandulosum* | 2 |
| Fabaceae | *Erythrina poeppigiana* |  |
|  | *Inga edulis* | 23 |
| Hypericaceae | *Vismia guianensis* | 13 |
|  | *Vismia sp.* |  |
| Malpighiaceae | *Byrsonima crassifolia* |  |
| Malvaceae | *Theobroma cacao* | 4 |
| Myristicaceae | *Virola sebifera* | 4 |
| Myrtaceae | *Eucalyptus sp.* |  |
|  | *Psidium guajava* |  |
|  | *Syzygium cumini* |  |
|  | *Syzygium malaccense* |  |
| Piperaceae | *Piper aduncum* |  |
| Polygonaceae | *Coccoloba latifolia* |  |
| Rhizophoraceae | *Rhizophora mangle* | 7 |
| Rubiaceae | *Isertia coccinea* |  |
| Rutaceae | *Citrus sp.* |  |
| Simaroubaceae | *Homalolepis cedron* |  |
| Urticaceae | *Cecropia obtusa* | 11 |

### Caterpillar models and predation marks

Artificial caterpillars (3 cm × 0.5 cm) were made from light green plasticine to mimic cryptic, palatable larvae. They were placed on branches 1.5–2 m above the ground and spaced at least 2 m apart along transects (100 per site, Fig. S2). After ~72h of exposure, caterpillars were collected and inspected for predation marks. Attacks were categorized visually based on impression shape, following published criteria (Eötvös and Lövei 2013; Lövei and Ferrante 2017; Fig. S3).

| 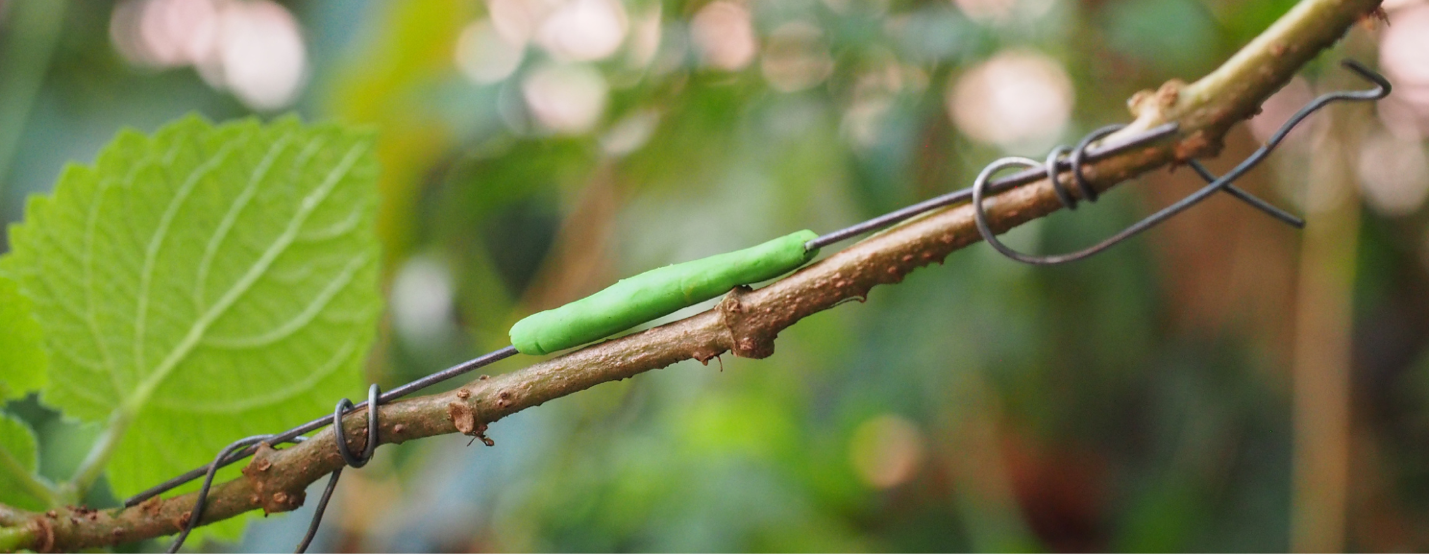 |
| --- |
| **Fig. S2** Photograph of artificial caterpillar placed on a branch |
| 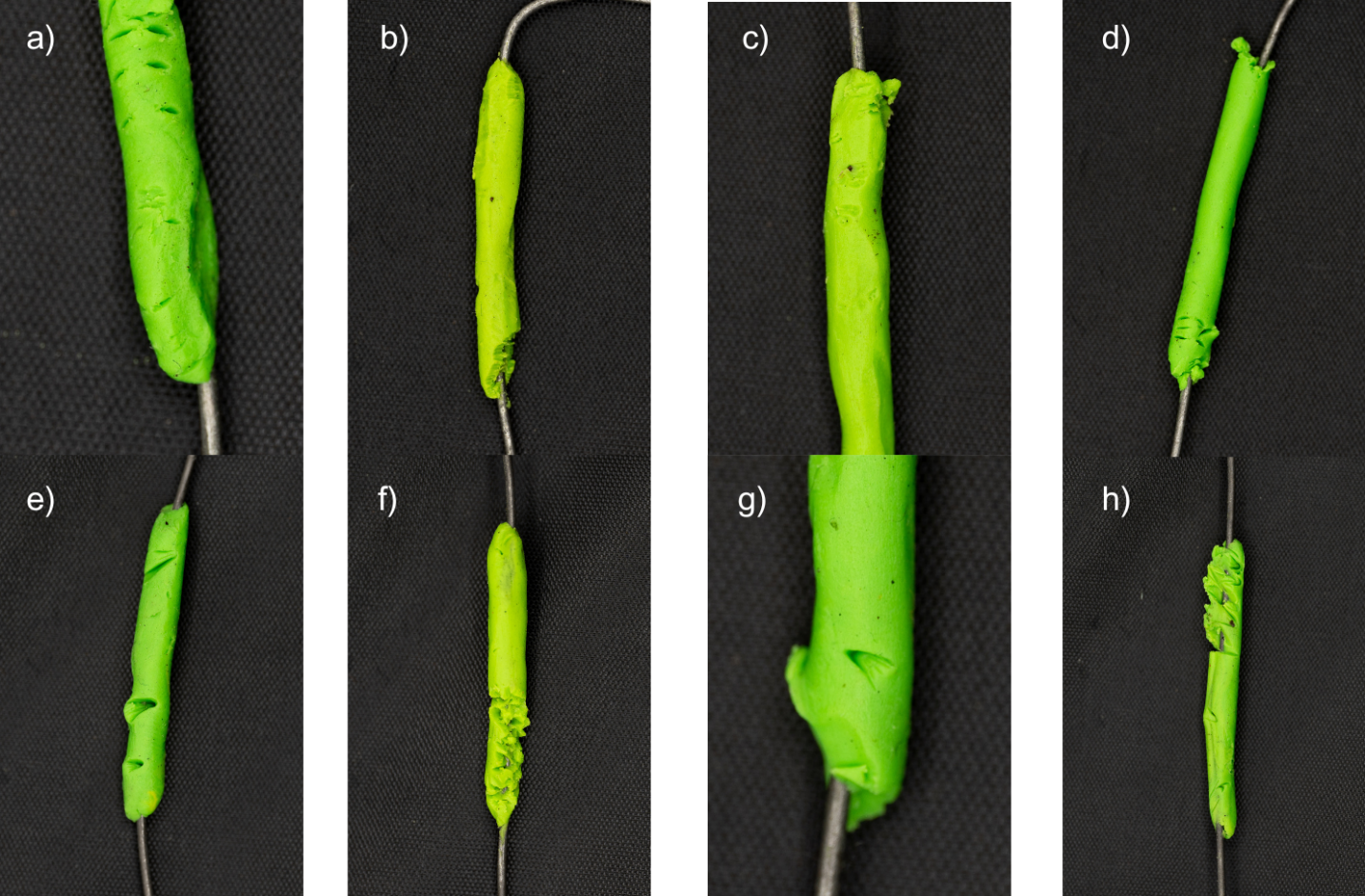 |
| **Fig. S3** Examples of predation marks left by: arthropods (a-d) and birds (e-h) |

###

### Variables used for the spatial generalized additive models

To assess habitat differences, spatial generalized additive models (sGAM) with ordinal response structure were used. Variables showing inconsistencies (e.g., total intercept measures and total intercepts for host plants), as well as those with a variance inflation factor (VIF) greater than 5, were excluded from the models (Table S3).

| **Table S3** Variables used for the proportional odds logistic regression models (POLR) analysis, with excluded variables (VIF > 5 or inconsistencies) indicated in bold. | |
| --- | --- |
| Variable | Description |
| Total basal area (m²) | The total basal area (sum of all Ge). |
| Basal area of host plants (m²) | The total basal area occupied by host plants (sum of all host plant Ge). |
| Canopy cover (%) | The tree canopy estimated based on photographs. |
| Number of host plants | The total number of host plant species that intercepted the transect. |
| Number of trees | The total number of trees that intercepted the transect. |
| Pielou’s evenness index (J') | A measure of species evenness within the transect, showing how evenly individuals are distributed across species. |
| Predation rate | Predation rate by birds on artificial clay caterpillars. |
| Species richness (S) | The total number of different tree species that intercept the transect. |
| **Basal area host plant ratio** | **The ratio of the total basal area occupied by potential host plants.** |
| **Intercept host plant ratio** | **The ratio of intercepts measured occupied by potential host plants.** |
| **Proportion of host plants** | **The proportion of trees that intercepted the transect that were potential host plants.** |
| **Shannon diversity index (H’)** | **Index that accounts for both species richness and the evenness of species abundance.** |
| **Total intercept measures** | **Sum of all intercept lengths.** |
| **Total intercepts for host plants** | **Sum of host plant intercept lengths.** |

### Evaluation of the spatial generalized additive model

Model assumptions were assessed using simulated residuals (DHARMa package). Deviation from uniformity (KS test: p = 0.65), overdispersion (p = 0.85), and outliers (p = 1) were not found. Moreover, no significant spatial autocorrelation was detected (Moran’s I = -0.14, p = 0.9). Predictive performance was evaluated using leave-one-out cross-validation and quantified using mean absolute error (MAE). The model showed limited predictive accuracy (MAE = 1.60), indicating substantial uncertainty in predicting outbreak categories for new sites, likely due to the small sample size and strong spatial structure of the data. Influence diagnostics identified two observations with elevated Cook’s distances. One corresponded to a transect in Mountain des Singes characterized by a combination of elevated host plant presence and high predation pressure relative to other transects at the same site, while the other was located in the savannah of Matiti and exhibited particularly low canopy cover (39%). Removal of these observations reduced model explanatory power and altered the significance of some predictors, particularly canopy cover. However, predation rate remained a significant predictor, indicating a robust effect across sites. These influential observations likely reflect ecologically distinct habitat conditions and contribute to the overall gradient structure of the dataset.

### Target-group background

To minimize biases in the model related to uneven spatial sampling, the target-group background method was applied. This approach refines the selection of pseudo-absences by focusing on areas where surveys similar to those conducted for our focal species have taken place. In this study, various moth families—Saturniidae, Sphingidae, and Noctuidae—were used as the target group for *H. metabus* (Fig. S4a), while the class Magnoliopsida was used as the target group for host plants (Fig. S4b).

| 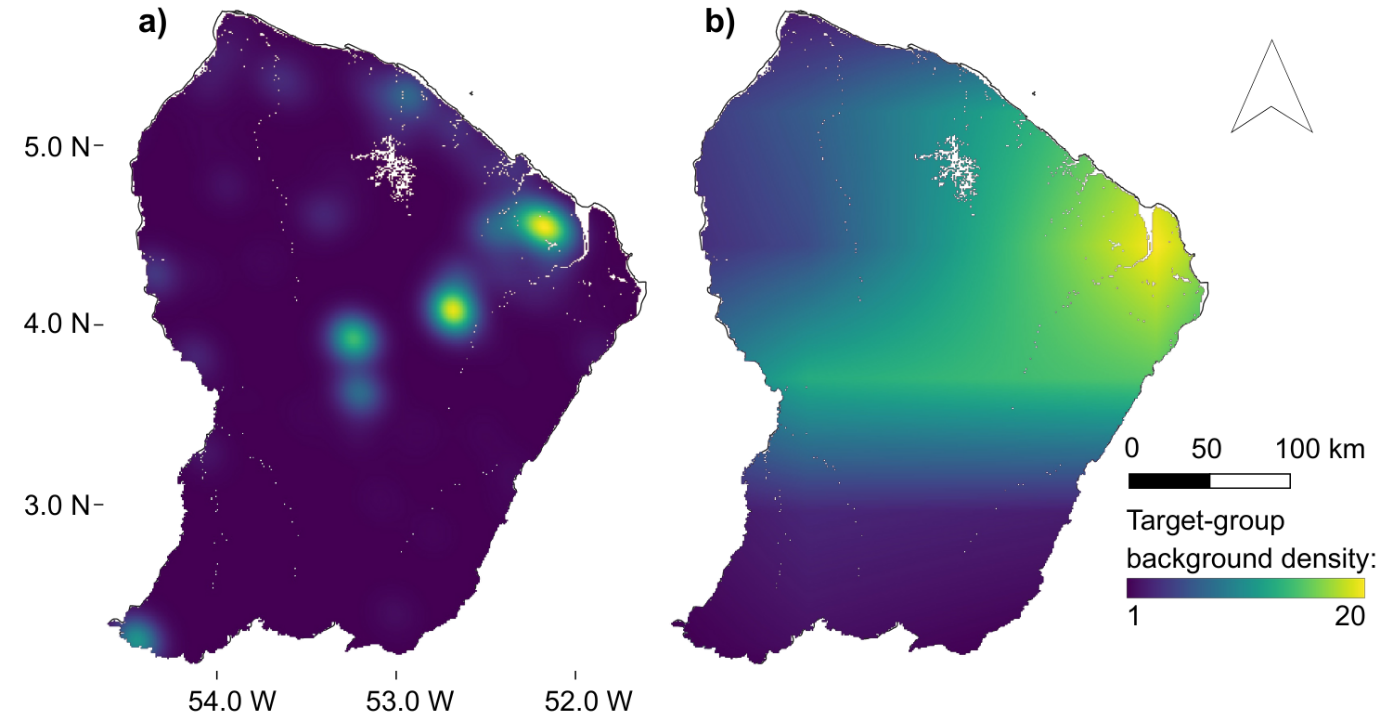 |
| --- |
| **Fig. S4** Kernel density maps of occurrence records used for the target-group background with (a) density of occurrences for moth families Saturniidae, Sphingidae, and Noctuidae, used as a target group for *Hylesia metabus*, and (b) density of occurrences for the plant class Magnoliopsida, used as a target group for host plants. Density values are scaled between 1 and 20 |

### Species distribution model evaluation

To select the optimal parameters for the species distribution models, we used the ENMeval R package (Muscarella et al. 2014), which allowed us to identify, for each species (*H. metabus* and the three host plants), the best combination of feature classes and regularization multiplier (RM). In order to ensure methodological consistency and facilitate comparative analyses between species, we applied the same selection procedure across all species (Table S4). The final models were calibrated using the same settings (feature classes = Linear + Quadratic, RM = 1), which resulted in ΔAICc values below 2 for all species, except for *Laguncularia racemosa*, where the selected model had a ΔAICc of 2.38. Nevertheless, this model was retained due to its high predictive performance (AUC = 0.94), which was considered acceptable for our analyses.

To assess the robustness of the species distribution models (SDMs), we conducted sensitivity analyses for each species by comparing models using a uniform random background (i.e., without sampling bias correction) and models excluding the most influential climatic variable (mean diurnal temperature range, Bio2). Model performance was evaluated using AUC, and spatial consistency between models was assessed using Pearson’s correlation between raster predictions. Across all species, models built with and without sampling bias correction showed highly similar spatial predictions (Pearson’s r ranging from 0.90 to 0.98), despite negligible or no differences in model performance (e.g., *Hylesia metabus*: AUC = 0.92 with and without bias correction). This indicates that accounting for sampling bias has a limited effect on the spatial patterns of predicted suitability. In contrast, excluding Bio2 resulted in a decrease in model performance for several species (e.g., *H. metabus*: AUC = 0.92 to 0.77; *Laguncularia racemosa*: 0.93 to 0.83), and reduced spatial congruence with the original models (r ranging from 0.45 to 0.85).

Overall, these results indicate that the presented SDMs are robust to assumptions regarding background sampling, but sensitive to the inclusion of key climatic variables. In particular, mean diurnal temperature range appears to play a central role in structuring the predicted distribution of *H. metabus*, while plant species distributions are comparatively less sensitive to its exclusion.

| **Table S4.** Results of ENMeval model tuning for *Hylesia metabus* and its host plants, showing the best-performing combinations of feature classes and regularization multipliers (RM). Models were ranked based on AICc, with ΔAICc values and AUC scores provided for each model. Models selected to ensure methodological consistency across species are shown in bold. | | | | | |
| --- | --- | --- | --- | --- | --- |
| **Species** | **Feature classes** | **Regularization multiplier** | **AICc** | **ΔAICc** | **AUC** |
| ***Hylesia metabus*** | LQ | 1.5 | 1159.42 | 0 | 0.92 |
|  | LQ | 2 | 1159.77 | 0.35 | 0.92 |
|  | LQ | 2.5 | 1160.18 | 0.76 | 0.92 |
|  | L | 1.5 | 1160.45 | 1.03 | 0.92 |
|  | LQ | 3 | 1160.65 | 1.23 | 0.92 |
|  | L | 2 | 1160.7 | 1.28 | 0.92 |
|  | LQHP | 3 | 1160.89 | 1.47 | 0.92 |
|  | L | 2.5 | 1161 | 1.58 | 0.92 |
|  | LQ | 3.5 | 1161.19 | 1.76 | 0.92 |
|  | LQH | 3.5 | 1161.19 | 1.76 | 0.92 |
|  | L | 3 | 1161.34 | 1.92 | 0.92 |
|  | **LQ** | **1** | **1161.4** | **1.98** | **0.92** |
| ***Avicennia germinans*** | LQ | 0.5 | 405.26 | 0 | 0.96 |
|  | L | 0.5 | 406.21 | 0.95 | 0.96 |
|  | **LQ** | **1** | **406.96** | **1.7** | **0.96** |
| ***Laguncularia racemosa*** | LQ | 2 | 209.85 | 0 | 0.95 |
|  | L | 0.5 | 210.4 | 0.56 | 0.93 |
|  | L | 1.5 | 210.44 | 0.6 | 0.95 |
|  | LQ | 2.5 | 210.89 | 1.04 | 0.95 |
|  | L | 2 | 211.82 | 1.97 | 0.95 |
|  | LQ | 3 | 212.09 | 2.24 | 0.95 |
|  | **LQ** | **1** | **212.23** | **2.38** | **0.94** |
| ***Tapirira guianensis*** | LQ | 0.5 | 807.79 | 0 | 0.76 |
|  | L | 0.5 | 807.97 | 0.18 | 0.76 |
|  | **LQ** | **1** | **808.15** | **0.36** | **0.76** |
|  | L | 1 | 808.23 | 0.44 | 0.76 |
|  | L | 4 | 808.47 | 0.68 | 0.76 |
|  | L | 1.5 | 808.57 | 0.78 | 0.76 |
|  | LQ | 1.5 | 808.64 | 0.85 | 0.76 |
|  | LQ | 3.5 | 808.79 | 1 | 0.76 |
|  | L | 2 | 808.99 | 1.2 | 0.76 |
|  | LQ | 2 | 809.24 | 1.44 | 0.76 |
|  | LQ | 4 | 809.44 | 1.65 | 0.76 |
|  | L | 2.5 | 809.49 | 1.7 | 0.76 |

## **Results**

### Mean values for all variables collected for each site

For each line-intercept transect, several forest structure and diversity indicators were recorded, including predation rate, tree abundance, total intercept length, basal area and canopy cover. Additionally, three diversity indices were calculated: species richness, Shannon diversity index, and Pielou’s evenness index. For each site, values were averaged across the three transects to obtain site-level means (Table S5). These site-level indicators were also averaged by habitat type—savanna, coastal forest, hill and valley forest and mid-altitude forest—to explore structural and compositional differences among habitat categories (Table S6).

### Avian predation rate

Of the 1,284 artificial caterpillars retrieved only 85 could be attributed to avian attacks, resulting in an average avian predation rate of 0.07. Although absolute predation rates were low overall, relative differences between sites were nevertheless observed, with values ranging from 0 to 0.17 (see Table S5 for means per localities). Moreover, the Kruskal-Wallis test did not detect a significant overall effect of habitat type on predation rates (Statistic = 6.25, p = 0.1). However, post-hoc Dunn’s test comparisons revealed significant differences between mid-altitude mountain forests and both coastal forests and savannas (statistic = -2.37, p = 0.02, statistic = -2.04, p = 0.04, respectively; Fig. S5), with predation rates being highest in mid-altitude forests (mean = 0.11). In contrast, predation rates were lowest in savannas (mean = 0.06) and coastal forests (mean = 0.04), although two sampled coastal forest sites exhibited marked variation (0.12 in white sand forests vs. 0 in mangroves). Predation rates in hill and valley forests were intermediate between those observed in coastal (savanna and coastal forest) and mid-altitude mountain forest, with an average predation rate of 0.09.

| 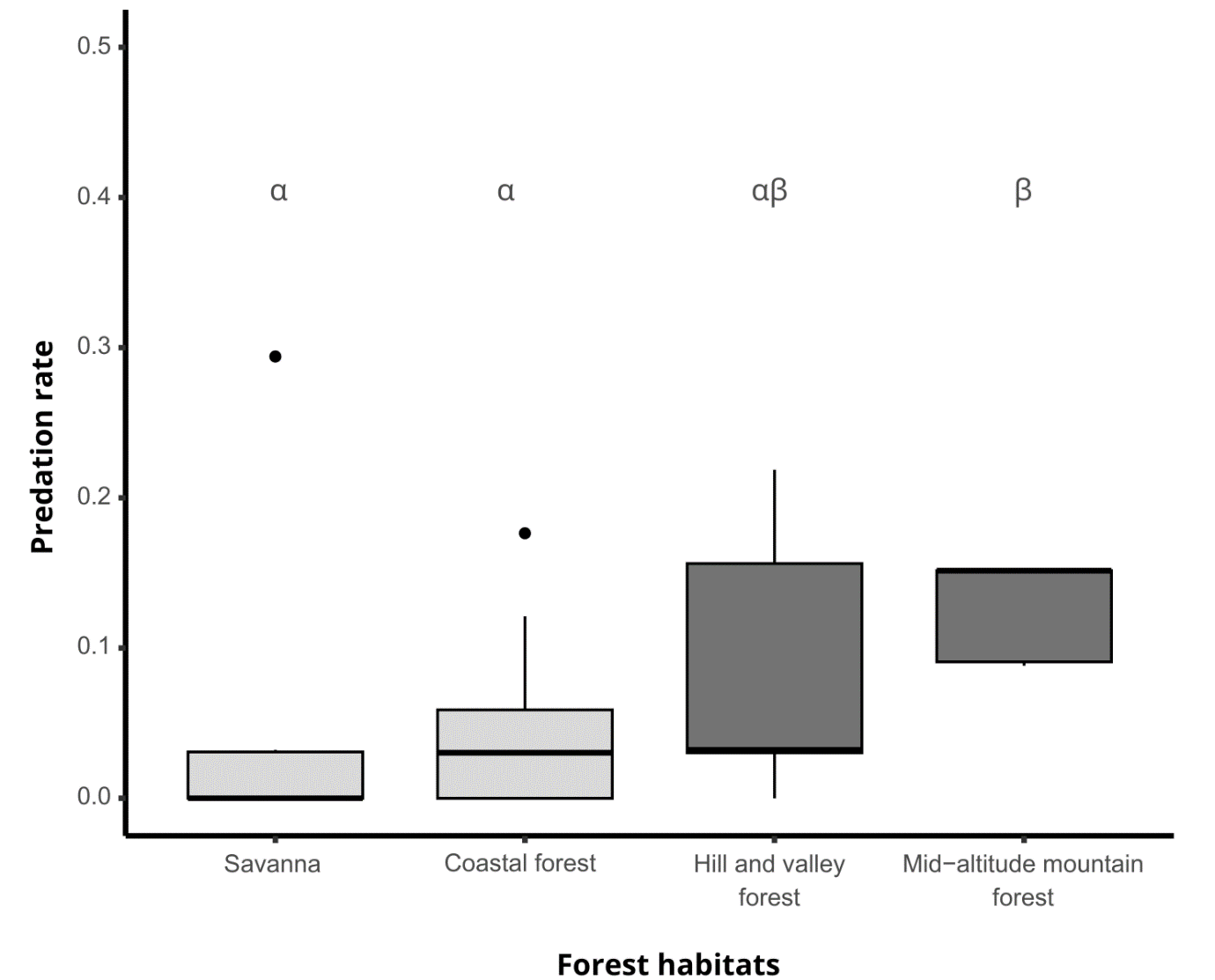 |
| --- |
| **Fig. S5** Differences in avian predation rates between savanna (N=4 sites), coastal forest (N=5 sites), hill and valley forest (N=2 sites), and mid-altitude mountain forest (N=2 sites). Coastal (light grey, including savannas and coastal forests) and inland (dark grey, including hill and valley forests and mid-altitude mountain forests) habitats are statistically different, as per the post-hoc Dunn’s test (α, αβ and β). |

### PCA

The principal component analysis (PCA) revealed that the first two axes explained 61.5% and 23.9% of the total variance respectively, capturing a cumulative 85.4% of the variability in the data. Predation rate and the number of trees were primarily associated with the two first axis (Predation rate: Dim1, loading = 0.75, contribution = 30%, cos² = 0.6; Dim2, loading = -0.60, contribution = 50%, cos² = 0.4; Number of trees: Dim1, loading = 0.74, contribution = 30%, cos² = 0.6; Dim2, loading = 0.60, contribution = 50%, cos² = 0.4). While the canopy cover was associated with the first and the third dimensions (Dim1, loading = 0.86, contribution = 40%, cos² = 0.7; Dim3, loading = -0.51, contribution = 60%, cos² = 0.3). Sites from Outbreak group 1 (least prone to outbreaks; *n* = 4) tended to show high canopy cover and tree richness (three sites with high positive Dim1 scores), with moderate to low predation rates (most sites close to 0 or slightly negative on Dim2). In contrast, Outbreak Group 2 (intermediate; *n* = 6) was more heterogeneous, including sites with both low and high tree richness and variable canopy cover, with sites relatively evenly distributed along both dimensions. Finally, Outbreak Group 3 (most outbreak-prone; *n* = 3) was characterized by lower canopy cover and tree richness (two sites with strongly negative Dim1 scores), and relatively high predation rates (two sites with positive Dim2 scores). These PCA findings are consistent with the results of the sGAM, which highlighted a decline in canopy cover and number of trees and an increase in predation rate along the outbreak gradient.

## *Species distribution model*

Species distribution models for *Hylesia metabus* and its host plants revealed a clear spatial pattern, with higher probabilities of occurrence concentrated along the coastline (Fig. S6). Importantly, all sampling sites included in this study were located within areas predicted to have relatively high probabilities of presence for each of the four species. This pattern was consistent even for sites where outbreak propensity was classified as low, suggesting that differences in spatial outbreak propensity are not simply driven by large-scale variation in climatic suitability.

| 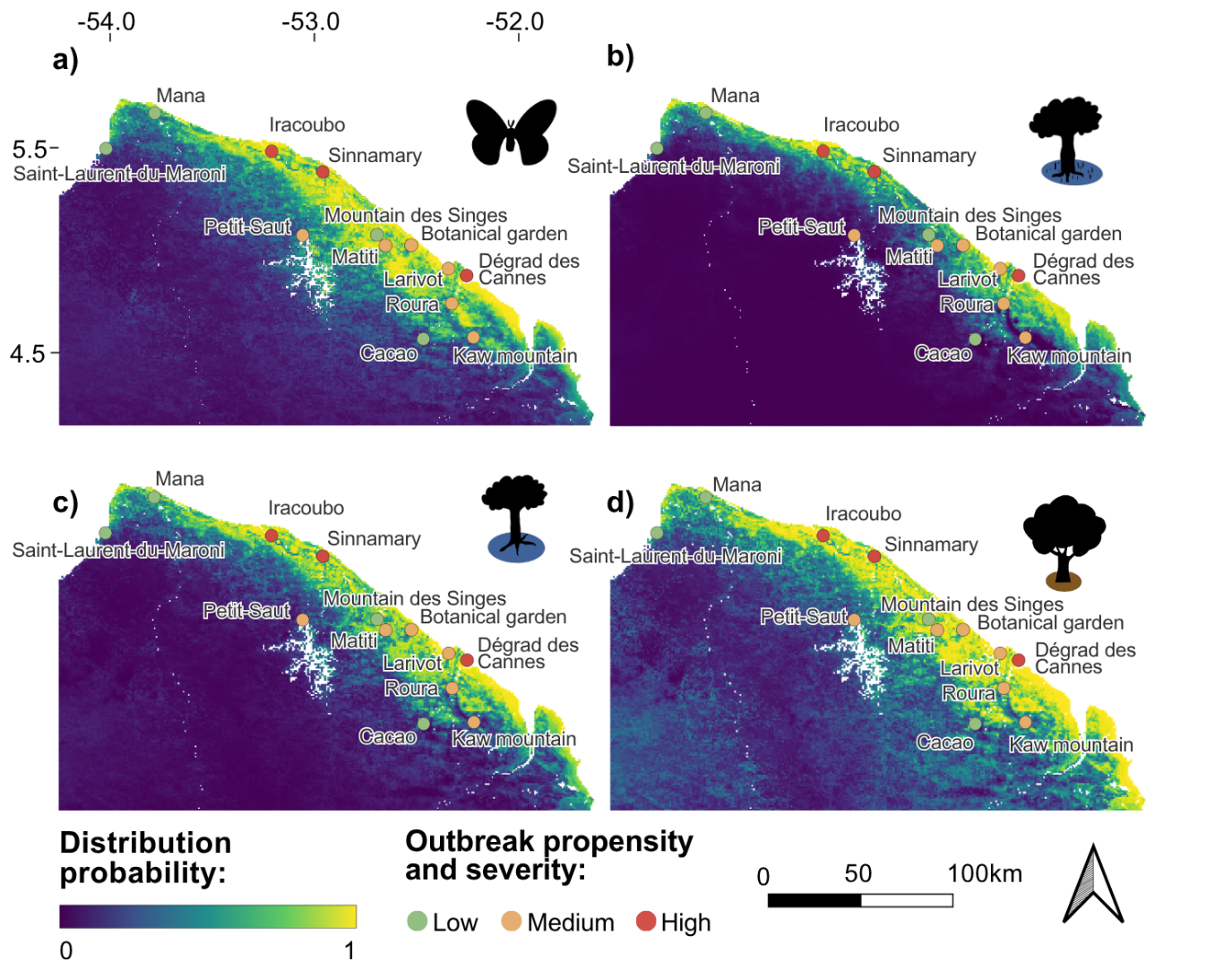 |
| --- |
| **Fig. S6** Species distribution models for (a) *Hylesia metabus*, (b) *Avicennia germinans*, (c) *Laguncularia racemosa*, and (d) *Tapirira guianensis*, along the coast of French Guiana. Predicted distribution probability ranges from 0 (blue) to 1 (yellow). The map also shows the spatial distribution of the 13 study sites, with colours indicating categories of *H. metabus* outbreak propensity: green denotes species presence with low numbers of reported outbreaks; yellow indicates a moderate number of outbreak reports, with occasional events causing mild impacts on human populations; and red indicates frequent and severe outbreaks, including widespread lepidopterism and the need for costly municipal interventions. |

| **Table S5** Mean values for all variables collected for each site; values equal to or superior to the overall median are in bold. | | | | | | | |
| --- | --- | --- | --- | --- | --- | --- | --- |
| Sites (with outbreak propensity in parentheses) | Predation rate | Number of trees | Number of host plants | Proportion of host plant | Total tree basal area (m²) | Basal area of host plants (m²) | Basal area host plant ratio |
| Cacao (1) | **0.07** | **11.33** | 2 | 0.16 | **3607** | 23 | 0.00 |
| Mountain des singes (1) | 0.01 | **12.00** | 0.33 | 0.05 | **1301** | 287 | 0.08 |
| Mana (1) | **0.12** | 6.00 | **2.67** | **0.48** | **1906** | **1217** | **0.36** |
| Saint-Laurent-du-Maroni (1) | 0.03 | **8.67** | **3.00** | **0.35** | **2807** | 258 | 0.22 |
| Lake Petit-Saut (2) | **0.17** | **13.00** | 0.33 | 0.04 | 1193 | 4 | 0.00 |
| Bridge Larivot (Macouria) (2) | 0.00 | 5.00 | **5.00** | **1.00** | **3369** | **3369** | **1.00** |
| Macouria botanical garden (2) | 0.01 | 5.33 | **3.33** | **0.56** | **1523** | **732** | **0.60** |
| Savannah of Matiti (Macouria) (2) | 0.01 | 6.00 | **2.33** | **0.39** | 897 | **590** | **0.71** |
| Roura (2) | 0.01 | **7.00** | **4.67** | **0.61** | 1129 | **376** | **0.61** |
| Kaw mountain (2) | **0.15** | **8.67** | **3.00** | 0.33 | 673 | **370** | **0.56** |
| Dégrad des Cannes (boat harbour, Rémire-Montjoly) (3) | **0.04** | **8.00** | **2.33** | 0.31 | **2378** | 166 | 0.09 |
| Iracoubo (3) | **0.11** | 5.67 | **2.33** | **0.38** | 571 | **294** | **0.53** |
| Sinnamary (3) | **0.12** | 6.33 | 1.00 | 0.15 | 1127 | 55 | 0.08 |
| **Median** | **0.04** | **7.00** | **2.33** | **0.35** | **1301** | **294** | **0.36** |
| Sites (with outbreak propensity in parentheses) | Total intercept measures | Total intercept for host plants | Intercept host plant ratio | Canopy cover (%) | Tree species richness (S) | Shannon diversity index (H') | Pielou's evenness index (J') |
| Cacao (1) | **2113** | **180** | 0.08 | **95** | **9.33** | **2.18** | **0.98** |
| Mountain des singes (Kourou) (1) | 53 | 10 | 0.11 | 89 | **8.33** | **1.73** | 0.92 |
| Mana (1) | **1623** | **887** | **0.55** | **94** | **4.67** | **1.35** | 0.90 |
| Saint-Laurent-du-Maroni (1) | **1747** | **870** | **0.49** | **96** | **5.33** | **1.56** | **0.95** |
| Lake Petit-Saut (2) | **1623** | 1 | 0.00 | **95** | **5.33** | 1.27 | 0.77 |
| Bridge Larivot (Macouria) (2) | 19 | 19 | **1.00** | 88 | 2.00 | 0.62 | 0.90 |
| Macouria botanical garden (2) | 24 | 15 | **0.64** | 68 | 2.67 | 0.87 | 0.90 |
| Savannah of Matiti (Macouria) (2) | 42 | 16 | **0.44** | 61 | **4.67** | **1.48** | **0.98** |
| Roura (2) | 42 | 20 | **0.55** | 79 | 3.67 | 1.31 | **1.02** |
| Kaw Mountain (2) | **1803** | **660** | **0.37** | **94** | **6.00** | **1.70** | **0.97** |
| Dégrad des Cannes (boat harbour, Rémire-Montjoly) (3) | **1463** | **497** | **0.36** | **97** | 4.33 | 1.30 | 0.89 |
| Iracoubo (3) | **1397** | **467** | **0.37** | 80 | 3.00 | 1.00 | **0.94** |
| Sinnamary (3) | **1460** | **193** | 0.13 | **95** | **5.33** | **1.56** | **0.93** |
| **Median** | **1460** | **180** | **0.37** | **94** | **4.67** | **1.35** | **0.93** |

###

| **Table S6** Mean values for some of the ecological variables and diversity indices measured per forest habitat for 13 sites in French Guiana. | | | | | | |
| --- | --- | --- | --- | --- | --- | --- |
| Habitat | Number of sites | Number of trees | Number of host plants | Proportion of host plants | Canopy cover (%) | Tree species richness (S) |
| Savanna | 4 | 5.83 | 2.25 | 0.37 | 76.07 | 3.92 |
| Coastal forest | 5 | 6.93 | 3.53 | 0.55 | 90.83 | 4 |
| Hill and valley forest | 2 | 12.50 | 0.33 | 0.04 | 92.10 | 6.83 |
| Mid-altitude mountain forest | 2 | 10 | 2.50 | 0.25 | 94.44 | 7.67 |

## **References**

Caratti JF (2006) Line Intercept (LI). In: Lutes DC, Keane RE, Caratti JF, Key CH, Benson NC, Sutherland S, Gangi LJ (2006) FIREMON: Fire effects monitoring and inventory system. Gen. Tech. Rep. RMRS-GTR-164-CD. Fort Collins, CO: U.S. Department of Agriculture, Forest Service, Rocky Mountain Research Station. p. LI-1-13. <https://doi.org/10.2737/RMRS-GTR-164>

CROPP (2015) La papillonite - Bulletin d’information n°10 Août 2015, Cellule régionale permanente d’observation et de prévention de la papillonite en Guyane

Eötvös C, Lövei GL (2013) Documenting predator marks on dummy caterpillars. In: Saska P, Knapp M, Honek A, Martinkova Z (eds) XVIth European Carabidologists Meeting - Book of abstracts with conference programme: carabids and man - can we live with(out) each other? European Carabidologists Meeting, p 31

Fraver S, Ducey MJ, Woodall CW, D’Amato AW, Milo AM, Palik BJ (2018) Influence of transect length and downed woody debris abundance on precision of the line-intersect sampling method. For Ecosyst 5:1–10

Lövei GL, Ferrante M (2017) A review of the sentinel prey method as a way of quantifying invertebrate predation under field conditions. Insect Sci 24:528–542

Muscarella R, Galante PJ, Soley-Guardia M, Boria RA, Kass JM, Uriarte M, Anderson RP (2014) ENMeval: An R package for conducting spatially independent evaluations and estimating optimal model complexity for Maxent ecological niche models. Methods Ecol Evol 5:1198–1205. https://doi.org/10.1111/2041-210X.12261

Oksanen J, Simpson GL, Blanchet FG, Kindt R, Legendre P, Minchin PR, O’Hara RB, Solymos P, Stevens MHH, Szoecs E, Wagner H, Barbour M, Bedward M, Bolker B, Borcard D, Carvalho G, Chirico M, Caceres MD, Durand S, Evangelista HBA, FitzJohn R, Friendly M, Furneaux B, Hannigan G, Hill MO, Lahti L, McGlinn D, Ouellette M-H, Cunha ER, Smith T, Stier A, Braak CJFT, Weedon J (2024) vegan: Community Ecology Package
